# Acceptability of HIV testing for men attending televised football venues in Uganda

**DOI:** 10.1186/s12889-019-7478-6

**Published:** 2019-08-19

**Authors:** Charles Peter Osingada, Godfrey Siu, Mathew Amollo, Patience Muwanguzi, Nelson Sewankambo, Noah Kiwanuka

**Affiliations:** 1 Department of Nursing, Makerere University College of Health Sciences, School of Health Sciences, P.O. Box 7072, Kampala, Uganda; 2 Makerere University Child Health and Development Centre, P.O Box 7072, Kampala, Uganda; 3 Department of Community and Environmental Health, Makerere University College of Health Sciences, School of Public Health, P.O Box 7072, Kampala, Uganda; 4 Department of Internal Medicine, School of Medicine, Makerere College of Health Sciences, P.O Box 7072, Kampala, Uganda; 5 Department of Epidemiology and Biostatistics, Makerere University School of Public Health, P.O Box 7072, Kampala, Uganda

**Keywords:** Acceptability, HIV testing, Men, Football, Uganda

## Abstract

**Background:**

Worldwide, HIV remains a major public health challenge, especially in Sub-Saharan Africa. Literature indicates that men’s involvement in HIV testing, care, and treatment services is lower compared to women, therefore novel approaches are required to engage men in the cascade of HIV care. This study aimed to explore men’s perception on the provision of HIV testing services in venues where English Premier League football games are televised.

**Methods:**

An exploratory qualitative study was conducted between February and May 2018. Six focus group discussions were conducted with 50 conveniently selected men aged 18 years and older using a pre-tested discussion guide. All focus group discussions were audio recorded, transcribed verbatim, and analyzed thematically.

**Results:**

Overall, HIV testing at venues telecasting English Premier League football games was acceptable to men. There was a very strong preference for health workers providing testing and counseling services be external or unknown in the local community. Possible motivators for testing services provided in these settings include subsidizing or eliminating entrance fee to venues telecasting games, integrating testing and counseling with health promotion or screening for other diseases, use of local football games as mobilization tools and use of expert clients as role models.

**Conclusions:**

This study suggests that HIV testing services at venues where EPL football games are televised is generally acceptable to men. In implementing such services, consideration should be given to preferences for external or unknown health workers and the motivating factors contributing to the use of these services. Given that HIV testing is currently not conducted in these settings, further research should be conducted to evaluate the feasibility of this approach as a means of enhancing HIV testing among Ugandan men.

## Background

Worldwide, HIV remains a major public health challenge especially in Sub-Saharan Africa (SSA). It is estimated that 36.7 million people are living with HIV globally with about 19.4 million of them living in Eastern and Southern Africa [1]. Widespread availability of Antiretroviral Treatment (ART) has led to a general decline in mortality associated with HIV. Nevertheless, the number of new infections remains unacceptably high particularly in resource-limited settings. In 2016, it was estimated that there were approximately 1.8 million new HIV infections equaling about 5000 new infections per day, with 64% of these infections occurring in Sub-Saharan Africa and mainly among women [1].

Although women in resource limited settings bear a high HIV burden, mortality associated with the disease is higher among men [2]. Studies indicate that men are less likely than women to be tested for HIV, are more likely to start ART in the later stages of HIV infection and also to be lost to follow-up which exposes them to a higher risk of experiencing early mortality [3–6]. Cognizant of this challenge the Joint United Nations Program on HIV/AIDS recommends several strategies for reaching out to men and boys as outlined in the Blind Spot report [7]. Similarly, the World Health Organization (WHO) “Consolidated Guidelines on HIV Testing Services” recommends a combination of facility and community- based approaches for reaching men; including provision of HIV testing services in appropriate and acceptable settings [8]. Studies have demonstrated that community-based HIV testing service approaches particularly mobile- testing, were more successful in reaching the highest proportion of men in SSA compared to facility-based testing modalities [9, 10]. However, concerns about linkage to care and sustainability, constrain community outreach programs.

In the Uganda population –based HIV impact assessment of 2017, HIV prevalence was reported as 6.2% among adults aged 15 to 64 years which corresponds to approximately 1.2 million people [11]. Similar to other Sub-Saharan countries [6, 12], involvement of men in HIV testing, care, and treatment services has been reported to be lower compared to women [13, 14]. This gender disparity may be linked to perceived fears of breach of confidentiality which can lead to stigmatization [15], perception of clinics as spaces for women [16],challenges associated with reputational masculinity [17] as well the perceived judgmental attitude of female health workers [16, 18]. The Uganda HIV Prevention and Treatment Guidelines recommend differentiated HIV testing services, delivery models including approaches aimed at engaging men such as outreaches at sporting events, workplaces, and key populations hotspots [19]. Anecdotal observations indicate that a section of Ugandan men both in rural and urban areas are ardent fans of televised English Premier League (EPL) football games and supporters of EPL clubs outnumber those of any other European league. Male fans spend long hours in bars, cinema halls and other venues where football games are televised, particularly on weekends. At the study sites, EPL football games were televised in venues that function only as places for broadcasting football games and viewing films, not as bars or drinking places.

The use of football as a vehicle to deliver health interventions for men has gained prominence in some developed countries, but this model has been tested in very few resource limited settings [20]. For example, in the United Kingdom, there have been interventions to promote physical activity, healthy eating and weight loss [21–24]. In South Africa, unemployed youth aged 18–25 years were recruited for soccer and job training to prevent drug abuse and HIV. They were tested for HIV and drugs and those who were successful were offered practical vocational training [25]. In Zimbabwe, a football-based intervention focused on secondary school students and it boosted medical male circumcision [20]. In Uganda, men patronize venues where EPL football games are televised which offers an ideal opportunity to mainstream health interventions targeting this sub-population. However, there is a scarcity of evidence to inform this model of service provision particularly in reference to HIV testing. In addition, differences engendered by culture and location limit the application of findings from developed countries to Uganda. Thus, this qualitative study aimed at eliciting men’s perceptions concerning provision of HIV testing services at venues where EPL football games are telecast. In adopting a qualitative approach, the investigators sought to gain a comprehensive understanding of men’s perspectives on HIV testing which will aid in developing an approach to increase HIV testing among men.

## Methods

### Study site and population

This exploratory qualitative study was conducted in three trading centers of Kumi District in Eastern Uganda. As Creswell (2013) suggests, this approach was suitable when investigators want to understand the issues in a natural context, and when the phenomenon does not lend itself to the traditional quantitative statistical approach [26]. The district has a projected population of 259,300 people, 127,000 of these are male [27]. Kumi District was purposively chosen as the study site because HIV testing services is disproportionately low among men compared to women. Of the 56,480 individuals aged 15 to 49 years who were tested for HIV in Kumi District in 2016/2017, only 31.1% were men and only 32% of those tested returned for their test results [28].

The study was conducted at Mukongoro, Kanyumu and Atutur trading centers, which were purposely selected because they are known to be HIV transmission “hot spots”. These sites are located more than five kilometers from each other. Only one football-televising venue from each trading center was included. Mukongoro trading center had three functioning venues, and one of the three was selected by a simple random method. Typically, an individual pays 500 to 1000 Uganda shillings (approximately 0.14–0.27 United States dollars) to watch a match depending on whether the football game involves top rated clubs or not.

### Selection of study participants

Participants in this conveniently selected sample were 50 men who came to watch an EPL football match on the day of participant recruitment. They participated in one of six focus group discussions (FGDs) The recruiters provided information about the study at each site before the scheduled time of the football match. Participants were included if they were males aged 18 years and older, current residents in the study area, fluent in Ateso (the language spoken in the study area), and football fans. Written informed consent was obtained from participants before each discussion commenced. They received the equivalent of a five US dollar honorarium to compensate them for their time. Two FGDs were conducted in each trading center; one with men aged 18–24 years, and another with men aged 25 years and older. In total 60 eligible men were invited to take part in FGDs but only 50 participated in the discussions.

### Data collection procedure

Data were collected from six focus group discussions each composed of 8–10 men. For Mukongoro and Kanyum trading centers, the FGDs were conducted privately in the halls were football games are usually televised. In Atutur trading center the FGDs were conducted in a church that was not in use. Data collection took 3 days; one day at each of the sites. Each discussion lasted between 30 min and 1 h. All the FGDs were conducted in Ateso (the local language spoken in the study area), using an FGD guide developed by the research team and translated into Ateso by a language expert from the Department of Linguistics at Makerere University. A moderator and a note taker conducted the FGDs. The FGD guide addressed a range of predetermined issues including acceptability of and motivators toward HIV testing at football venues as well as preferences of which health care personnel should be involved in testing. Data collection was an interactive process. The investigators listened to audio recordings of the first two group discussions in order to identify gaps that needed to be addressed in subsequent FGDs. Data collection stopped after six FGDs because no new information emerged from the interactions.

### Data management and analysis

Data were transcribed verbatim and then transcripts were translated into English. The transcripts were reviewed individually for completeness and accuracy and any text with participant identifiers was removed to maintain anonymity. The investigators adapted a descriptive content approach, as outlined by Sandelowski [29],to describe the perceptions of the men, the social context and discern meanings from the different voices reflected in the data. Data analysis was conducted following the parameters of: perceived acceptability of HIV testing at places telecasting premier league football, health care personnel, and motivators for HIV testing. To distill narratives, context and meanings from the transcripts, data was organized into emerging themes. A codebook was developed after reading through the first two transcripts, and this informed the preliminary sub-themes that were refined as other transcripts were coded and new themes identified. NVivo11 Pro was used in the analysis and coding of data. Two independent qualitative researchers reviewed the codes before consensus was achieved. The coding structure for each theme was reviewed after a preliminary analysis of a sub-sample of transcripts, and the codebook refined through comparison and categorization of its properties and dimensions.

## Results

### Participants’ characteristics

Fifty men of unknown HIV status participated in the study. The median age was 24.5 years, with a range from 19 to 71 years. Slightly over half of the participants were single and most of them earned a living through subsistence cultivation and livestock rearing. Additional information about the social demographic characteristics of the participants is in Table 1.

**Table 1:** Social demographic characteristics of FGD participants

Variable	Number	Percentage
Age		
19–24	25	50.0
25–71	25	50.0
Marital status		
Single	27	54.0
Married	23	46.0
Level of education		
Primary	21	42.0
Secondary	22	44.0
Tertiary	7	14.0
Religion		
Protestant	20	40.0
Catholic	12	24.0
Pentecostal	10	20.0
Muslim	8	16.0
Occupation		
Peasant	29	58.0
Businessman	7	14.0
Commercial motorcycle rider	3	6.0
Driver	6	12.0
Manson	2	4.0
Teacher	3	6.0

The men provided a variety of insights into their perceptions regarding HIV testing at sports entertainment venues that were categorized and presented in three major thematic areas of perceived acceptability, personnel, and motivators.

### Perceived acceptability

The general perception across the focus groups was that HIV testing at venues telecasting EPL games would be acceptable to men. Some of the participants expressed their views as follows:

*I really support the idea of testing from this place like this one here (football venue) because sometimes in other places like in the hospital when you go to test people will begin gossiping and talking in low voices “today that one has also come here to test, today he has arrived, what could be the issue”? (FGD-03)*

*I support that idea would work because always here in the halls (where they watch football) if you look at the population out there, then you see the age groups that is always common in the hall (watching football) you find they are the group that is very active sexually, the very strong ones are here watching football, it is rare for people who are elderly.(FGD-03)*

*It is a good idea because it helps you to know the status of your blood (HIV status) and also general health so that people will not just get surprised next year that so and so has died, but instead you will be able to go for treatment early.(FGD-01)*

The participants cited many reasons for supporting the testing for HIV at these venues, including the possibility of reaching many young men who are sexually active, the perception that testing at these venues is less stigmatizing, and the desire to have HIV services brought closer to where the men are. The opinions expressed highlighted men’s underlying concern for discretion and convenient access to HIV testing, as the following quotes illustrate:

*It will make some people happy because some have not been able to go and test their blood if the places for testing blood are very far, now when it comes to a place like here, it will find him around. (FGD-02)*

*It’s better testing here in the football venue, you look there at the hospital (pointing at a nearby hospital), you see that house which is isolated on its own, [laughter], they call it “for the sick” (HIV positive), that means the moment one steps there or just going near it, it means you are sick (HIV positive) [laughter]. (FGD-01)*

*If it is brought in places such as this (football venues) we shall also be able to find time to come and test but if it is in the market places, if you are tested and found to be sick (HIV positive) you fear like they will just tell openly that you are sick (HIV positive) and everyone there will begin laughing at you. (FGD-02)*

### Preferences regarding health personnel

The FGDs also covered aspects regarding professional behavior, age, and gender of the healthcare personnel deemed appropriate to provide HTC services at the football venues. This thematic area was derived from three categories; confidentiality, trust and gender preference.

#### Confidentiality

Participants from all six focus groups emphasized the need to have professional health workers who exhibit professional behavior such as confidentiality, to conduct the HIV testing at the EPL telecasting venues.

*The doctors who come to conduct the testing should have the knowledge not to spread peoples results because if the ones of this place get to know that one of them was tested and he is HIV positive and the doctor is the one spreading that information, when they go to another hall, I don’t think they will accept to be tested. (FGD-02)*

#### Trust

The men did not appear to trust the ability of health workers from local health facilities to keep HIV test results confidential. A common thread that weaved through all the focus groups was a deep-seated fear that health workers from within the community, and who knew them may not keep the HIV test results confidential, and feared the resulting stigma in the event of a positive result. Fear of being tested by health workers who knew them and their families well, reportedly discouraged many men from testing and encouraged them to lie about having already been tested for HIV. In a focus group involving older men, it was voiced repeatedly and vehemently that most men would not come for HIV testing and would falsely state that they had been tested for HIV if health workers known to them were recruited to conduct HIV testing in their community.

*Most of the medical workers here they know us, after they have carried out the test they go and begin telling people, you see that one, he has HIV, I tested him, they talk in form of fun, ((in whisper)) the son of so and so has been tested HIV positive. (FGD-02)*

*The person who should be assigned to handle has to be trusted, because here when you go to test, someone who knows you can decide to deceive you and give you wrong results. (FGD-03)*

Due to concern related to trust, many men expressed the need to travel long distances to places where they were not known in order to take an incognito HIV test.

*Most men, according to my simple research, which I have tried to carry out, they mostly, go to far places such that if they found themselves to be finished, (HIV positive) they come back peacefully and keep quiet. (FGD-01)*

Thus, for HIV testing services to be accepted by men at local televised football entertainment venues, health workers from distant places were the preferred providers.

*They [referring to health workers] should not be people from within but outside, say like people from …, but if you bring people from within…, or within the district no one will accept to test. Everyone will tell you that for me I have finished to test [laughter]. (FGD-01)*

*It is better to bring someone from far, may be from … who will not go on spreading information on peoples results for example if I am tested positive (HIV positive) he will not go and talk about me in the drinking joints because he doesn’t know me.(FGD-02)*

Cases of community conflicts involving a particular health provider were also reported to lower the level of trust that men had in the health workers based in their community health facilities. For example, cases of failed relationships and land ownership issues undermined trust, resulting in questioning of the HIV services provided by such a health worker irrespective of the level of professionalism demonstrated.

*Sometimes may be because you have befriended his daughter [laughter] he finds you, he gives you false results. (FGD-02)*

#### Gender preference

There were mixed views on the preferred gender of health professionals who provide HIV testing services. The data indicated that both male and female health workers would be accepted. The group that preferred female health workers argued that women were better caregivers who could easily interact with males compared to fellow men. They argued that the females would be better suited in the area of counseling:

*To me ladies are good when it comes to counseling even if you are dead* [referring HIV positive] *they help to restore your heart to peace. (FGD-01)*

*Women will tell you the truth, they will not deceive you, that’s why I want women to be the ones to test other than men. (FGD-03)*

*you need to have a bigger number of females, since the target is to test men, that’s when you will realize bigger numbers turn up, you bring the “chicks*”[referring to young females]*…with some who are there just only to talk, you will realize a great result, but if you bring men, people will refuse to test. (FGD-01)*

However, the group that preferred male health care workers for HIV testing claimed that women are emotional, and may not cope well with the emotional stress associated with a positive HIV test result in their client. Other reasons identified for preference of a male provider were, desire of some men to start relationships with the same female health workers, thereby compromising their efficiency, while others suggested that men might not easily open up to a woman:

*No, no, no, it should be men, with girls you might meet again somewhere may be in a school and you try to win her to love. (FGD-01)*

*It will not be fitting for a woman to carryout testing, first because a woman is emotional, a woman when she meets a fellow woman can tell her issues (gossip), a woman may fail to tell her husband issues yet she is telling them to another woman in her neighborhood. (FGD-03)*

*They will just struggle for women to test them because this very people who are being tested want to ask for love from the same women and they talk with them as they carry out the tests. (FGD-02)*

#### Age perception

Participants below 25 years of age indicated that they would be encouraged to test more if the health providers were closer to them in age. They perceived that more youthful health workers would understand their interests and challenges as opposed to those who are older.

*I think it would be good to bring the youth like our age mates, they are the ones people like these days such that if I come to test like from my brother here, I can tell him that please kindly help me and give me my results alone when it is only me who knows my status. (FGD-01)*

#### Perceptions of HIV diagnosis

HIV is still associated with death with participants expressing fear of HIV positive results. The results show that communities still stigmatize HIV patients and therefore, to avoid the community wide stigma, some men prefer to seek HIV testing services from health facilities where they are not known or opt to obtain HIV diagnosis kits and self-test for HIV.

*Most men, according to my simple research, which I have tried to carry out, they mostly go far off places such that if they found themselves to be finished, (HIV positive) they come back peacefully and keep quiet. (FGD-01)*

*There those they sell in the clinics, someone goes secretly and asks for the procedure how they conduct the test, and after they tell him he goes and does the test, after testing he doesn’t know the results he just goes and keeps quite. FGD-01-25+*

The practice of HIV self-testing is predominately among the youth. This emerged in all group discussions of men aged more 25 years.

*Some especially youths who are educated go and buy their testing kit mostly from the clinic, because they are sold in clinics, they come with their local pad, bleeder, those things… then he comes and tests himself in the house (at home). FGD-02-25+*

### Motivators

Several strategies to motivate men to participate in HIV testing services in sports viewing venues were identified. These included cost related incentives and service integration strategies:

#### Subsidizing or eliminating venue entrance fees

All six focus groups proposed that reducing or eliminating entrance fees charged to watch televised matches would dramatically increase use of HIV testing services at these venues.

*Since these days people fear that watching football is expensive it would be better to tell them these two days we are going to undertake blood testing for HIV so we shall watch football for free, the most important thing is go and test your blood for HIV and you go straight to watch football. (FGD-02)*

*Tell them that today everyone will watch for free, yet there is a condition for you to watch for free, then when they ask what is that condition, then they tell them that you are supposed to do a blood test, but you don’t tell them that it is for HIV, you tell them some simple, simple disease. (FGD-01)*

*If it is free entry to watch, the whole of this trading center will come, the hall may even become too small. (FGD-01)*

*what could be done is to pay the owner of that video hall for all the time to be taken while running that activity [when he has been given his cash], so that the customer just keeps coming to watch and go away, watch and go away just like that. (FGD-01)*

*If there was money, the best way to attract this people would be to tell them that these days there is an organization sponsoring all the football matches, then you watch for free, for whatever number of people, the organization will pay you just enter to watch. You would see that this house is full to capacity; it might even require to be enlarged. (FGD-03)*

#### Multi-disease screening and deception

The participants also stated that there would be more willingness to utilize HIV testing services if this was coupled with screening for other ailments such as Hepatitis B, Syphilis, and Brucellosis. During the discussions, the researchers informed the participants that this would be a form of deception that is unethical and increases mistrust towards healthcare workers especially those working in the area of HIV.

*It is better to tell them they are testing for other diseases like brucellosis, like that because there are very many people who have not tested for brucellosis compared to hepatitis B, while for you who carryout testing you know what you are testing for HIV. (FGD-01)*

*You first don’t tell them they are testing for HIV, tell them we are testing for hepatitis, they will come very many, now after they have arrived and one is called to the small room then you tell them that there is testing for hepatitis but also let us test for HIV and we shall not hang your results just, the results will be delivered at your home. (FGD-03)*

*That is when you will now get them properly, don’t even talk about HIV, just talk about all the diseases in your body will be tested and it is free of charge. (FGD-01)*

#### Using local friendly football matches to attract men

It also emerged that local friendly matches in locations where EPL televising venues are found can be a powerful tool for mobilizing men for HIV testing. Such events would be officiated by local or national football heroes, who would rally their supporters to not only attend the matches but also take the test.

*For us here in … we like football so much if there is a way you could come and call for a friendly match. Or you call a team from … and the one here … then you announce that on such and such a day there is going to be a football match, such a day people will be very many. (FGD-01)*

*Inform the people (fans) that we shall have our visitors coming, they are from this particular teams or they can be told that there is a manager of Eastern region Federation of Ugandan Football Association (FUFA) Cup from somewhere in … the fans will be told that our managers who are in charge of the whole of Eastern Region are coming here with all fans, those on ManU with theirs of Man U, Arsenal with theirs of Arsenal like that, all those people (fans) will come in bigger numbers early.(FGD-02)*

#### Using expert clients and peers

The role of expert clients and peers in mobilization for participating in HIV testing services emerged from two of the six focus group discussions.

*You tell them you see that one his health condition was bad but when he tested and discovered, he has greatly improved because he has accepted to take up the medication. (FGD-02)*

*Some will accept to test because their friend has also tested, because they will say if I don’t test my friend will think that eh, this man has refused to test does it mean he knows he is dead (HIV Positive). (FGD-02)*

## Discussion

The aim of this study was to explore men’s perception on the provision of HIV testing services in venues where English Premier League football games are televised. The findings suggest that provision of HIV testing services at venues where EPL games are televised is generally acceptable to men. There was a very strong preference for health workers who are external to the community to provide HIV testing and counseling services. Possible motivators for HIV testing provided in these settings included subsidizing or eliminating entrance fees to venues telecasting games, integrating testing and counseling with health promotion or screening for other diseases.

With regard to acceptability, findings from this study resonate with evidence already reported by other studies indicating that community-based HIV testing interventions have higher acceptability than health care facility based approaches [30, 31]. Thus, given the popularity of EPL among men in the rural and urban settings of Uganda and other Sub-Saharan African countries, these findings provide an indication for evaluating the feasibility of mainstreaming health interventions such as HIV testing and counseling in entertainment venues. There have been efforts in Australia to test the feasibility of using football as a conduit to assess the possibility of conducting screening for Sexually Transmitted Infections (STIs) among young men in rural football clubs. If proved successful, interventions provided in non-medical settings, particularly in the area of HIV, have the potential to reduce the gender gap in HIV testing, treatment and care services [32].

Encouraging individuals to seek HIV testing and counseling services from the nearest health facility did not emerge from this study. Participants in all six focus groups emphatically stated a preference for health workers from distant places and those not known in the local community to conduct the testing. This preference seems to stem from concerns regarding confidentiality. Similar findings have been reported in Tanzania where some participants indicated a strong preference against a counselor they knew or who resides in the same neighborhood [33]. Participants from the Tanzanian study also opted to travel far from their communities to obtain HIV testing services. This implies that even if HIV testing and care services are brought closer to where individuals live, it may not lead to utilization of the service as long as there is mistrust between health workers and clients [34]. The phenomenon of individuals traveling farther to access HIV care services has also been reported by Akullian et al. (2016) but in this case it was construed to be driven by limited availability of specialized HIV services and preference for higher level healthcare facilities [35]. However, there is evidence that such behavior may result from a need to avoid stigmatization from members of the immediate community [36, 37].

Results of this study further highlight the positive role incentives could play in increasing use of HIV testing services in venues telecasting EPL. Finding from this study are in contrast to a study by Njau et al. (2014) in which some participants indicated that payment to individuals could be used to get more people tested [33]. In the present study, participants suggested that subsidizing or eliminating entrance fees to venues where football games are televised would potentially increase attendance of the televised games and may also result in increased use of HIV testing services. The role of incentives in positively influencing health behavior has been highlighted in previous studies [38–41]. With respect to HIV testing, incentivized programs demonstrated a positive effect in increasing use of HIV testing services in both adult men [42–44] and in children and older adolescents [45]. However, incentivized approaches so far tested involve provision of monetary rewards, redeemable vouchers or lottery opportunities for individuals to win money. Findings from this study provide an opportunity of testing the effect of abolishing entrance fees to football venues on the use of HIV testing services offered at venues telecasting EPL. Because this model does not depend on direct provision of money to participants, it may provide an opportunity for repeat HIV testing. This might address some of the limitations plaguing community-based HIV screening services. However, the design of such a study would still need to take into consideration concerns of equity in regard to gender and socioeconomic status, coercion, and other ethical challenges which arise in relation to using incentives to modify health behavior [41, 46]. A non-monetary incentivized HTC service approach at these venues coupled with the rapidly growing concept of HIV self-testing [44], holds promise in optimizing HIV testing among men.

In this study, participants pointed out the importance of having HIV testing services coupled with screening for other infections and diseases. Feasibility of integrated health campaigns in reaching a large population with health prevention interventions has been demonstrated in previous studies [47, 48]. Indeed a multi-disease testing approach has previously been reported to yield very high HIV testing coverage at low marginal costs [49]. A study conducted in rural Uganda and Kenya employing a hybrid mobile approach to multi-disease community health campaigns achieved a high HIV testing coverage [50]. While this approach is capable of reaching a substantial number of individuals in the community, in most cases men tend to be under-represented compared to women. Nevertheless, application of the concept of multi-disease screening in venues televising EPL has the potential of reaching many men while reducing the stigma associated with HIV testing alone. In EPL venues, health campaigns could be linked with screening for tuberculosis and prostate cancer both of which are common conditions among men.

Participants also recommended that men should be lured to test under false pretenses that they are screening for other diseases yet they are being tested for HIV. This proposal is clearly unethical and increases mistrust of health providers in HIV care. An on-going and trusting patient-provider relationship is one of the most important contributors to the health of people living with HIV and the way this relationship starts at testing will determine the way a person progresses along the continuum of HIV care [51].

In this study it also emerged that friendly local football games could be used as a mobilization strategy to attract men and this would possibly increase utilization of HIV testing services. This finding is consistent with analysis by Mwaanga (2010) that sport can be used as bait to attract young participants to provide core HIV services such as voluntary HIV testing and HIV life skills education [52]. In a related study Clark and colleagues observed an increase in HIV related knowledge among students who participated in an education program delivered by role models who were professional soccer players [53]. While participants in this study did not directly allude to the critical role models play in championing sports-based health interventions, Tobisch and Preti (2010) noted that sports ambassadors can be important in promoting HIV prevention messages [54], a role also demonstrated by an evaluation study conducted by Clark and others [53].

Finding of this study also underscore the role of expert clients and peers in encouraging men to take an HIV test. Previous studies have documented the importance of integrating expert clients in HIV services provision. For example, in Zimbabwe linkage of HIV positive clients to care within 7 days was significantly associated with the period of engaging expert clients in the project [55]. In Uganda expert clients have helped to increase uptake of DNA-PCR testing among infants [56], and medical male circumcision services [57]. Therefore, involvement of HIV positive clients as champions in community sports-based HIV testing services may yield good results.

### Limitations

This study was not without limitations. First, this study did not investigate why football venues in particular resonate with men, how football shapes the identity, masculine norms, and how these combine to influence men’s access to HIV testing. Delving into these aspects would have generated interesting information that would have enriched this study. Thus, further research is needed to illuminate these areas. Second, the authors did not share the data with participants in order to confirm that their views were captured accurately and this might have compromised the trustworthiness of the findings. However, every measure was taken during transcription to ensure the original views of the participants were not distorted.

## Conclusion

The study data suggests that HIV testing services at venues where EPL football games are televised is generally acceptable to men. In implementing such services, due consideration should be given to preferences for external or unknown health workers and motivators for use of the services. Given that HIV testing is currently not conducted in these settings, further research should be conducted to evaluate the feasibility of this approach as a means of enhancing HIV testing among Ugandan men.

## Data Availability

The datasets used and/or analyzed during the current study are available from the corresponding author on reasonable request.

## Abbreviations

AIDS: Acquired Immune Deficiency Syndrome
ART: Antiretroviral Therapy
DNA: Deoxyribose Nucleic Acid
EPL: English Premier League
FGD: Focus Group Discussion
HIV: Human Immuno-deficiency Virus
HMIS: Health Management Information System
HTC: HIV Testing and Counseling
HTS: HIV Testing Services
IRB: Institutional Review Board
PCR: Polymerase Chain Reaction
UNCST: Uganda National Council for Science and Technology
WHO: World Health Organization
